# Population pharmacokinetic analysis of lopinavir in HIV negative individuals exposed to SARS-CoV-2: a COPEP (COronavirus Post-Exposure Prophylaxis) sub-study

**DOI:** 10.1186/s40360-023-00687-6

**Published:** 2023-09-27

**Authors:** Paul Thoueille, Margot Delfraysse, Pascal Andre, Thierry Buclin, Laurent A. Decosterd, Chiara Fedeli, Pilar Ustero, Alexandra Calmy, Monia Guidi, the Swiss HIV Cohort Study

**Affiliations:** 1 Service and Laboratory of Clinical Pharmacology, Department of Laboratory Medicine and Pathology, Lausanne University Hospital and University of Lausanne, Lausanne, Switzerland; 2 Division of Infectious Diseases, Faculty of Medicine, Geneva University Hospitals, Geneva, Switzerland; 3 Department of Medicine, Faculty of Medicine, University of Geneva, Geneva, Switzerland; 4 Centre for Research and Innovation in Clinical Pharmaceutical Sciences, Lausanne University Hospital and University of Lausanne, Lausanne, Switzerland; 5 Institute of Pharmaceutical Sciences of Western Switzerland, University of Geneva, University of Lausanne, Geneva, Switzerland

**Keywords:** Lopinavir, Pharmacometrics, COVID, Therapeutic drug monitoring, NONMEM

## Abstract

**Background:**

Lopinavir/ritonavir (LPV/r) is a drug traditionally used for the treatment of HIV that has been repurposed as a potential post-exposure prophylaxis agent against COVID-19 in the COronavirus Post-Exposure Prophylaxis (COPEP) study. The present analysis aims to evaluate LPV levels in individuals exposed to SARS-CoV-2 versus people living with HIV (PLWH) by developing a population pharmacokinetic (popPK) model, while characterizing external and patient-related factors that might affect LPV exposure along with dose–response association.

**Methods:**

We built a popPK model on 105 LPV concentrations measured in 105 HIV-negative COPEP individuals exposed to SARS-CoV-2, complemented with 170 LPV concentrations from 119 PLWH followed in our routine therapeutic drug-monitoring programme. Published LPV popPK models developed in PLWH and in COVID-19 patients were retrieved and validated in our study population by mean prediction error (MPE) and root mean square error (RMSE). The association between LPV model-predicted residual concentrations (C_min_) and the appearance of the COVID-19 infection in the COPEP participants was investigated.

**Results:**

A one-compartment model with linear absorption and elimination best described LPV concentrations in both our analysis and in the majority of the identified studies. Globally, similar PK parameters were found in all PK models, and provided close MPEs (from -19.4% to 8.0%, with a RMSE of 3.4% to 49.5%). No statistically significant association between C_min_ and the occurrence of a COVID-19 infection could be detected.

**Conclusion:**

Our analysis indicated that LPV circulating concentrations were similar between COPEP participants and PLWH, and that published popPK models described our data in a comparable way.

**Supplementary Information:**

The online version contains supplementary material available at 10.1186/s40360-023-00687-6.

## Background

Since the beginning of the COVID-19 pandemic, great efforts have been made to develop effective and safe vaccines active against SARS-CoV-2, as well as antiviral drugs to prevent infection or reduce symptoms and/or complications. In that context, lopinavir combined to ritonavir (LPV/r), a drug traditionally used for the treatment of HIV, has been repurposed as a potential post-exposure prophylaxis (PEP) agent against COVID-19 within the COPEP (COronavirus Post-Exposure Prophylaxis) open-label cluster randomized controlled superiority trial conducted in Switzerland and in Brazil between March 2020 and March 2021 [1]. Four randomized clinical trials published prior to the COPEP study did not show any effect on clinical endpoints when the antiviral was administered during hospitalization for severe COVID-19 [2–5]. However, LPV/r was never tested in early stages or as a PEP strategy. The COPEP study demonstrated that LPV/r over 5 days did not significantly reduce the incidence of COVID-19 in exposed individuals, however without completely ruling out a possible role for LPV/r in PEP, which remains to be confirmed or refuted.

The present study was not designed to estimate the impact of established COVID-19 on the pharmacokinetics (PK) of LPV/r, which has been already demonstrated elsewhere [6]. Instead, it aimed to evaluate LPV levels in individuals participating in the COPEP trial compared to people living with HIV (PLWH), by developing a population PK (popPK) model, while characterizing external and patient-related factors that could affect LPV exposure along with dose–response association.

## Methods

### Study population

Individuals exposed to SARS-CoV-2 more than 15 min at less than 2 m distance or having shared a closed space for more than 2 h with a person with confirmed SARS-CoV-2 infection were enrolled. Dried blood spots (DBS) were obtained from capillary puncture in participants included in Geneva, Basel and Lugano (Switzerland) after they received LPV/r (400/100 mg) twice daily for 5 days as PEP. Sampling was performed on day 5, and the time of last drug intake was carefully documented. Exclusion criteria for the present popPK analysis were LPV blood concentration below 1000 ng/mL (i.e., 12 concentration measurements were removed from the analysis), considered specific for absolute non-adherence to PEP, and non-reliable information on time and/or date of last drug intake and/or blood collection. Demographic factors, clinical information and comedications were available for the analysis.

LPV was quantified using a multiplex liquid chromatography coupled to tandem mass spectrometry method previously developed in the Laboratory of Clinical Pharmacology (CHUV, Lausanne, Switzerland) [7] and adapted for DBS levels quantification (Supplementary Data).

### Population pharmacokinetic analysis

The non-linear mixed effects modelling software NONMEM® (v7.4.3, ICON Development Solutions, Ellicott City, MD, USA) was used for the popPK analysis. Data management, graphical exploration and statistical analyses were performed with R (v4.0.2, R Development Core Team, http://www.r-project.org/).

DBS concentrations obtained during COPEP study were transformed into plasma concentrations according to the following equationn [8–10]:

$${C}_{plasma}= \frac{{C}_{DBS} \times {F}_{BP}}{1 -HCT}$$

where C_plasma_ is the plasma concentration (ng/mL); C_DBS_ is the blood concentration measured in the DBS samples (ng/mL); F_BP_ is the LPV protein binding ratio (98.5%) [11]; HCT is the volunteer haematocrit value, set at 0.4 for women and 0.45 for men because this information was not collected in the COPEP study [8]. Population pharmacokinetic analysis was performed on these COPEP plasma-converted data complemented with sparse plasma concentrations obtained from PLWH enrolled in the Swiss VIH Cohort Study (SHCS; http://www.shcs.ch) and followed up in the routine therapeutic drug monitoring (TDM) programme of the Service of Clinical Pharmacology in Lausanne (Switzerland) between January 2010 and May 2022. All individuals were considered at steady state, assuming full treatment adherence for the COPEP participants and long treatment duration for the SHCS patients.

### Models development, evaluation and assessment

A classic stepwise procedure [12] allowed identifying the popPK base model that best fit LPV data from the COPEP study and the routine TDM programme, and the sources of variability through a forward insertion/backward deletion approach. The following covariates were tested, using linear or allometric functions as deemed appropriate: age, sex, bodyweight, height, body mass index, smoking status and type of population (i.e., COPEP vs PLWH). The latter was investigated on all the PK parameters. Covariate analysis of concomitant medications was not performed because no reported medications were susceptible to drug-drug interactions with LPV/r. Hierarchical models were statistically discriminated at a significance level of 0.05 in forward model building (ΔOFV < -3.84 for one additional parameter) and of 0.01 in backward deletion (ΔOFV > 6.63 for the removal of one parameter) steps. Finally, the accuracy of PK parameter estimates and model shrinkage, as well as goodness-of-fit diagnostic plots, informed model selection and assessment of the reliability of the results. Prediction- and variability-corrected visual predictive checks (pvcVPCs) were performed on the final PK model to compare the observed concentrations with the 5^th^, 50^th^, and 95^th^ prediction percentiles [13–15], whereas the boostrap method (*n* = 2000) [13] contributed to model evaluation by comparing the original model estimates to the bootstrap median parameter values and their 95% confidence intervals (CI_95%_).

In addition, external validations of published LPV/r popPK models (identified by the following research equation: ("Lopinavir"[Mesh]) AND ("population pharmacokinetics*"[tiab] OR "population pharmacokinetic analysis*"[tiab] OR "population pharmacokinetic model*"[tiab] OR "popPK"[tiab]) NOT ("Child"[Mesh] OR "Infant"[Mesh] OR "Pregnant Women"[Mesh] OR "Pregnancy"[Mesh] OR "Tuberculosis"[Mesh] OR “Rifampicin”[tiab])) were performed by fixing their popPK parameters to the estimated values and comparing log-transformed concentrations and predictions with mean prediction error (MPE) and root mean square error (RMSE) to quantify model’s accuracy and precision, respectively.

### Association between LPV drug exposure and the occurrence of SARS-CoV-2 infection

The association between in-house popPK model log-transformed predicted LPV trough levels (C_min_) at day 5 (i.e. at the end of the PEP period) and the occurrence of SARS-CoV-2 infection at day 21 in the subset of patients with baseline negative test was investigated through a one-way analysis of variance (*p* = 0.05) to complement our initial analysis of the role of LPV/r exposure on the incidence of COVID-19 in exposed individuals [1].

## Results

Regarding the COPEP study, 105 participants contributed to 105 LPV concentration (i.e., one DBS each). On the other hand, 170 LPV sparse plasma levels from 119 PLWH were retrieved from the TDM programme database. Table 1 and Figure S1 (Supplementary Data) present the characteristics of the study populations and the observed concentrations, respectively.

**Table 1:** Characteristics of the participants

Baseline characteristics	COPEP population (*n* = 105)	PLWH (routine TDM) (*n* = 119)
	**Median (range) or Number (%)**	**Median (range) or Number (%)**
Demographic characteristics		
Sex (no.):		
Male	45 (43)	50 (42)
Female	60 (57)	69 (58)
Age (year)	39 (17—67)	41 (19—78)
Body weight (kg)	73 (47—157)	66 (40—147)
Height (cm)	172 (149—192)	168 (148—190)
BMI (kg/m^2^)	24 (17—49)	24 (16—51)
Drug and sampling		
Lopinavir dosing (mg):		
200	-	9 (8)
300	-	1 (1)
400	105 (100)	85 (71)
500	-	5 (4)
600	-	7 (6)
800	-	10 (8)
1000	-	2 (2)
Number of samples per patient	1 (1—1)	2 (1—8)
Time after dose (h)	1.5 (0.07—19)	10.25 (1.25—29.5)

*PLWH* People living with HIV, *TDM* Therapeutic drug monitoring, *BMI* Body mass index

### Structural, statistical and covariate models

In line with the popPK models found in the literature (Table S1) [16–21], a one-compartment model with first-order absorption and elimination best described LPV plasma concentrations. PopPK parameters between COPEP participants and PLWH did not differ significantly, thus supporting the use of a unique model for both COPEP and TDM data. Base model parameter estimates were a first-order absorption rate (k_a_) of 0.743 h^−1^, a volume of distribution (V_LPV_) of 78.9 L and a clearance (CL_LPV_) of 4.05 L/h, with an associated inter-individual variability (IIV) of 30%. The assignment of IIV on V_LPV_ and k_a_ did not improve data description (ΔOFV = 0, *p* > 0.05). A combined error model best described LPV residual unexplained variability. Univariate analyses revealed a significant linear association between bodyweight and CL_LPV_ (ΔOFV = -13.2, *p* < 0.001), with a 19% higher CL_LPV_ in a person of 100 kg vs 70 kg. Allometric scaling described equally well the effect of weight on CL_LPV_, but the linear function was retained for simplicity.

### Models evaluation and validation

**Fig. 1:**
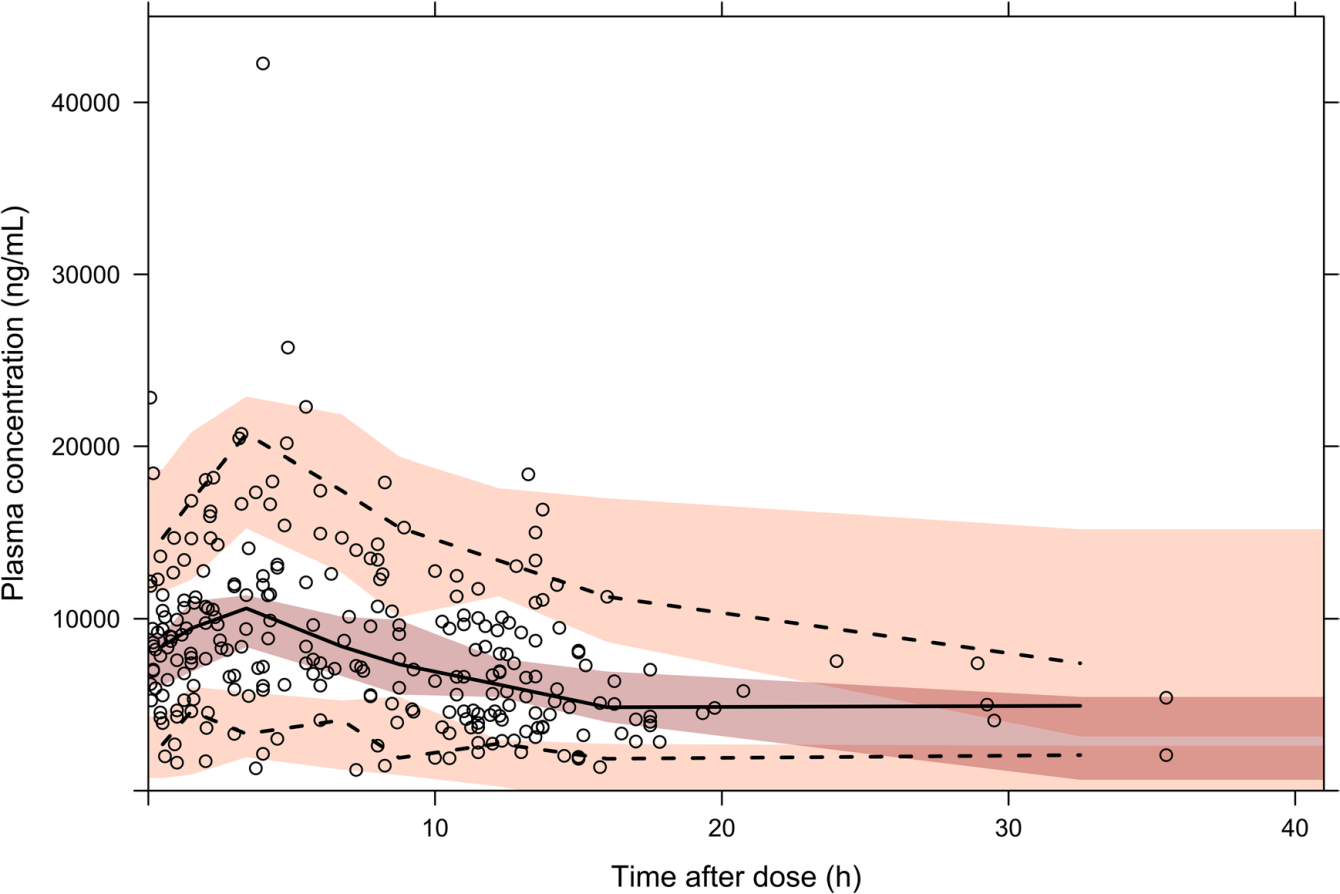
Visual predictive check of the final in-house model for LPV. Open circles represent the observed plasma concentrations; black solid and dashed lines represent the median and PI_90%_ of the observed data, respectively; shaded surfaces represent the model-predicted 90% confidence interval of the simulated median and PI_90%_. Note: One concentration with time after dose beyond 40 h is not displayed

**Fig. 2:**
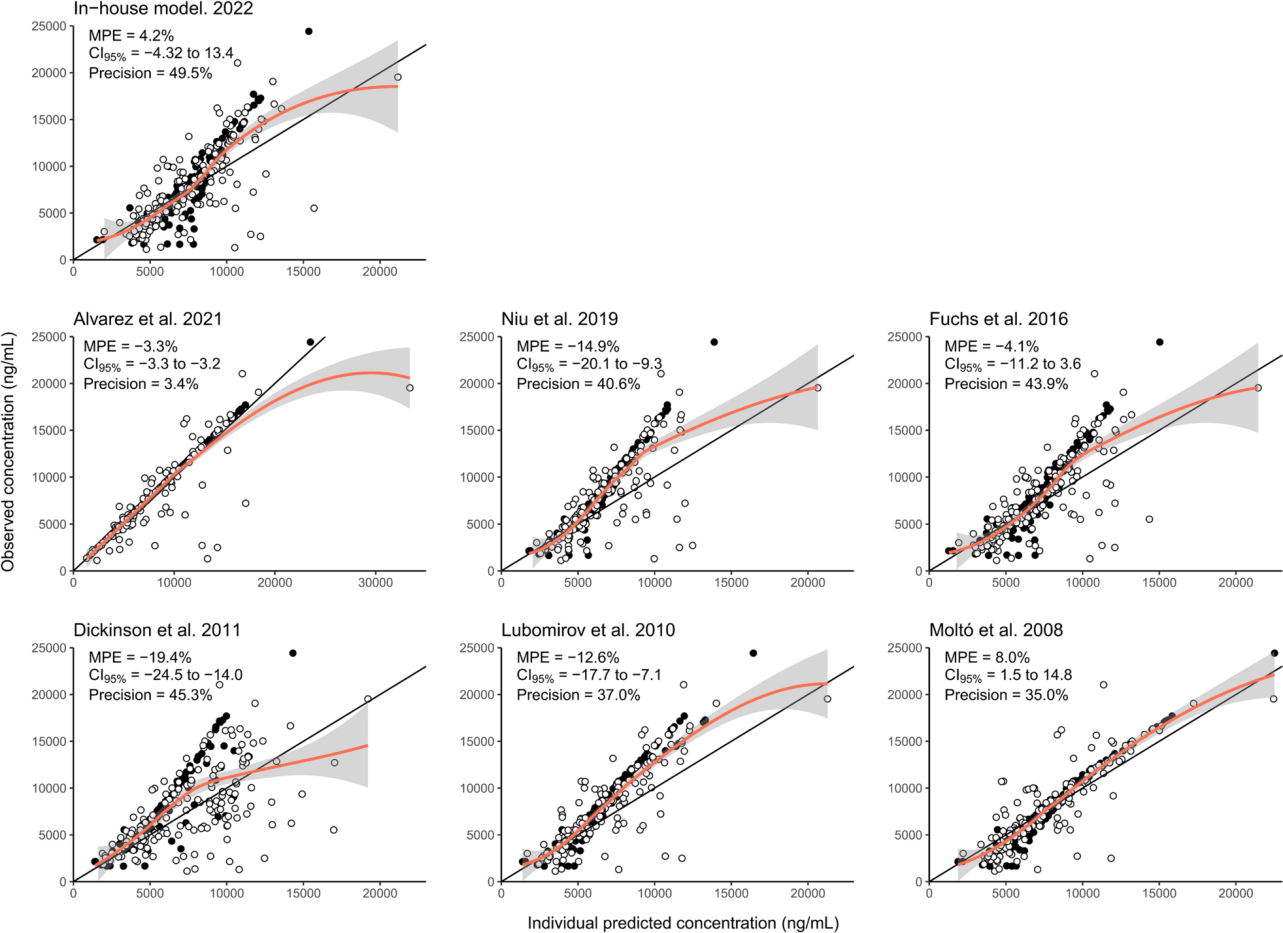
Assessment and evaluation of models performance. The black lines illustrate the identity lines, while the orange lines represent the local polynomial regression fits and the grey areas their 95% confidence intervals. The black points show the data from COPEP trial, and the white points illustrate data from the routine TDM. MPE: mean prediction error; CI_95%_: 95% confidence interval

Bootstrap (Table 2) and pvcVPC results (Fig. 1) confirm the good performance of our model. Figure 2 shows the individual predictions obtained from the in-house and different published popPK [16–21] models on our data, while Table S1 summarizes the corresponding popPK parameters. Globally, the PK parameters found in the different studies were similar to those obtained with our popPK model, and provided similar MPE (minimum of -19.4% to a maximum of 8.0%, with a precision (RMSE) of 3.4% to 49.5%).

**Table 2:** Final population parameter estimates of LPV with their bootstrap evaluations

Parameters	Final model				Bootstrap (*n* = 2000 samples)			
	**Estimate**	**RSE (%)**^**a**^	**BSV (%)**^**b**^	**RSE (%)**^**a**^	**Median**	**CI**_**95%**_^**c**^	**BSV (%)**^**b**^	**CI**_**95%**_^**c**^
k_a_ (h^−1^)	0.76	3			0.75	0.37—66.41*		
V_LPV_ (L)	78.9	2			77.7	52.2—119.5		
CL_LPV_ (L/h)	4.02	3	28.5	14	4.00	3.71—4.32	28.0	18.8—35.9
θ_BW_	0.447	14			0.447	0.196—0.733		
σ_prop_ (%)	33.3	11			32.7	23.0—39.6		
σ_add_ (ng/mL)	1560	1			1544	1005—2247		

Final model:

$${TVCL}_{LPV} = {CL}_{LPV}*\left(1+ {\theta }_{BW}*\frac{BW-70}{70}\right)$$

*k*_*a*_ first-order absorption rate constant, *V*_*LPV*_ apparent volume of distribution of lopinavir, *CL*_*LPV*_ apparent clearance of lopinavir, *θ*_*BW*_ bodyweight effect on *CL*_*LPV*_ with reference bodyweight of 70 kg, *σ*_*prop*_ proportional residual error; σadd: additive residual error

^a^Relative standard error (RSE) of the estimate defined as SE estimate/estimate, expressed as a percentage, with SE estimate retrieved directly from the NONMEM output file

^b^Between-subject variability

^c^95% confidence interval

*See Figure S2 for illustration of the CI_95%_ estimated for k_a_ after bootstrapping

### Simulations

**Fig. 3:**
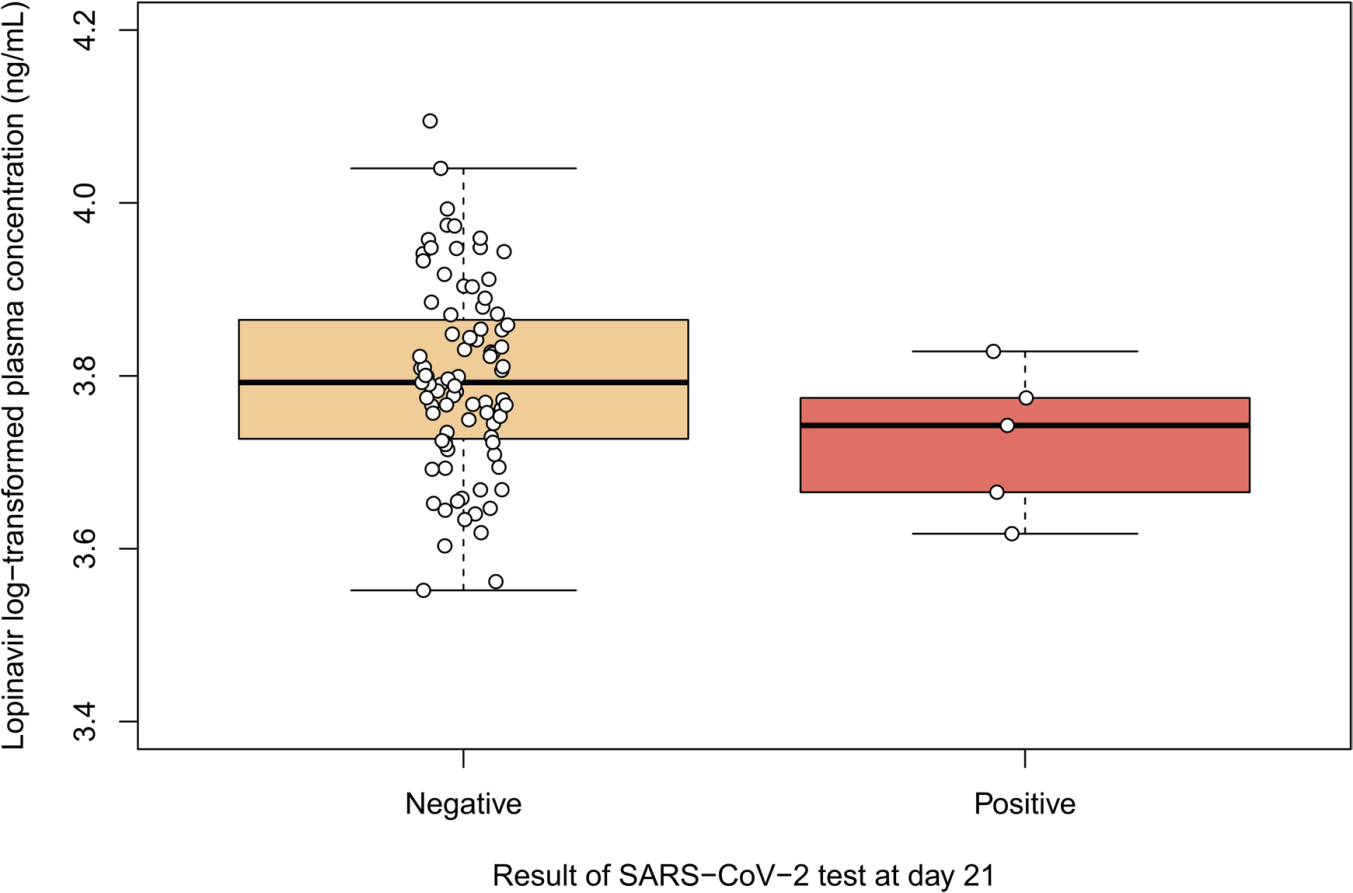
Individual prediction of LPV log-transformed plasma trough concentration (C_min_, white points) after 5 days of post-exposure prophylaxis pooled according to the SARS-CoV-2 test result 21 days after the exposure. Log-transformation of C_min_ values was applied to improve the normality of this positively skewed parameter

Figure 3 presents the log-transformed individual prediction of LPV plasma C_min_ at day 5 pooled according to the SARS-CoV-2 test result 21 days after exposure. No clear association between log-transformed LPV C_min_ at day 5 and occurrence of a positive test to SARS-CoV-2 was statistically found (*p* = 0.142).

## Discussion

Our popPK analysis showed that a one-compartment model with linear absorption and elimination best described LPV concentrations, which were similar between COPEP participants and PLWH enrolled in the SHCS. The present study showed that the different published popPK models performed broadly similarly in describing our data, with the exception of the one developed by Dickinson et al*.*, and thus that LPV PK is essentially comparable in various populations [19]. LPV T_max_ derived using the in-house base model was 3.9 h, in fair accordance with the official monography [11], and the half-life (t_1/2_) of 13.5 h was similar to the values obtained from the published popPK models (Table S1) [16–21].

Our analysis was limited by the COPEP study design not primarily conceived for a popPK analysis (e.g., patients’ self-reported information) with a unique DBS sample often collected close to drug administration. The enrichment of the COPEP dataset with TDM concentrations increased the informativeness of the data mostly in the elimination phase, and allowed for a better description of inter-individual and residual unexplained variabilities. However, there was still a relatively high shrinkage of 37% on CL_LPV_ IIV, rendering the use of classic diagnostic plots of reduced value [22]. Keeping this in mind, an overall tendency to underestimate high concentrations, especially the COPEP trial concentrations (black points, Fig. 2), was observed for all models except those developed by Alvarez et al*.* [16] and Moltò et al*.* [21]. In addition, the hematocrit assumption (i.e., set at 0.4 for women, and at 0.45 for men) may have affected the conversion of lopinavir DBS to plasma concentrations, possibly preventing the identification of other factors influencing lopinavir disposition.

Finally, the lack of significant association between Bayesian-extrapolated C_min_ and the occurrence of a positive SARS-CoV-2 test does not bring new hints regarding correlation between LPV concentrations and the hazard to develop the infection, already reported as not significant in the comprehensive analyses performed in the princeps paper [1].

## Conclusion

In conclusion, our analysis showed that circulating LPV concentrations were similar between COPEP participants and PLWH. Published popPK models overall described our data in a comparable way.

### Supplementary Information

**Additional file 1.** Dataset analyzed during this study.

**Additional file 2: Figure S1.** Observed plasma concentrations of LPV for the two study populations. **Figure S2.** Distribution of k_a_ estimated by bootstrap. **Table S1.** PK parameters of the published popPK models.

## Data Availability

The dataset generated and analyzed during the current study is available in a supplementary file.

## Abbreviations

CI_95%_: 95% Confidence intervals
CL_LPV_: Clearance of lopinavir
C_min_: Trough concentration
DBS: Dried blood spots
IIV: Inter-individual variability
ka: First-order absorption rate
LPV/r: Lopinavir/ritonavir
MPE: Mean prediction error
OFV: Objective function value
PEP: Post-exposure prophylaxis
PK: Pharmacokinetics
PLWH: People living with HIV
popPK: Population pharmacokinetics
pvcVPCs: Prediction- and variability-corrected visual predictive checks
RMSE: Root mean square error
t_1/2_: Half-life of elimination
TDM: Therapeutic drug monitoring
V_LPV_: Volume of distribution of lopinavir
