# Supplementary material for: Population pharmacokinetic analysis of lopinavir in HIV negative individuals exposed to SARS-CoV-2: a COPEP (COronavirus Post-Exposure Prophylaxis) sub-study
Source: BMC Pharmacol Toxicol. 2023 Sep 27;24:47. doi: 10.1186/s40360-023-00687-6 (PMC10536696; doi:10.1186/s40360-023-00687-6)
Supplement: Supplementary file 2 — Additional file 2: Figure S1. Observed plasma concentrations of LPV for the two study populations. Figure S2. Distribution of ka estimated by bootstrap. Table S1. PK parameters of the published popPK models. [file 40360_2023_687_MOESM2_ESM.docx]

**Evaluation of the pharmacokinetic profile of lopinavir in HIV negative individuals exposed to SARS-CoV-2: a COPEP (COronavirus Post-Exposure Prophylaxis) sub-study**

Paul THOUEILLE ^1^, Margot DELFRAYSSE ^1^, Pascal ANDRE ^1^, Thierry BUCLIN ^1^, Laurent A. DECOSTERD ^1^, Chiara FEDELI ^2^, Pilar USTERO ^2^, Alexandra CALMY ^2,3^, Monia GUIDI ^1,4,5^*, and the Swiss HIV Cohort Study

^1^ Service and Laboratory of Clinical Pharmacology, Department of Laboratory Medicine and Pathology, Lausanne University Hospital and University of Lausanne, Lausanne, Switzerland

^2^ Division of Infectious Diseases, Geneva University Hospitals, Faculty of Medicine, Geneva, Switzerland

^3^ Department of Medicine, Faculty of Medicine, University of Geneva, Geneva, Switzerland.

^4^ Centre for Research and Innovation in Clinical Pharmaceutical Sciences, Lausanne University Hospital and University of Lausanne, Lausanne, Switzerland

^5^ Institute of Pharmaceutical Sciences of Western Switzerland, University of Geneva, University of Lausanne, Geneva, Switzerland

*Corresponding author: Monia Guidi, +41 21 314 38 97, [Monia.Guidi@chuv.ch](mailto:Monia.Guidi@chuv.ch)

Running title: Lopinavir population pharmacokinetics

# Supplementary Data

## Concentration determination

Briefly, the mobile phases (i.e. H_2_O + 0.2% formic acid (A) and acetonitrile (B)) were delivered at flow rate of 0.3 mL/min on a 2.1 x 75 mm C18 silica-based column (Xselect 3.5 µm HSS T3, Waters, Milford, MA, USA) using the following gradient: isocratic gradient at 2% B for 1 minute, followed by a linear gradient from 2% to 30% B in 2.2 minutes, up to 100% B in 6.8 minutes. Subsequently, solvent B was reduced to 2% (initial conditions) in 0.2 minutes, followed by a re-equilibration step up to 12 minutes (total analysis time). The chromatographic system was coupled to a TSQ Quantiva triple quadrupole mass spectrometer from Thermo Fisher Scientific, equipped with an Ion Max NG^TM^ electrospray ionization source. The limit of quantification of the method was 10 ng/mL for LPV.

The extraction procedure of DBS samples was as follows: a 3 mm disc was punched out from the centre of DBS samples and mixed with 100 μL of internal standard solution (LPV-d8 at 2500 ng/mL in methanol). After the tubes were sonicated for 10 minutes and centrifugated (10 minutes at 14’000 rpm and 4°C), 70 μL of supernatant were diluted with 100 μL of H_2_O milliQ. The injection volume was 20 μL.


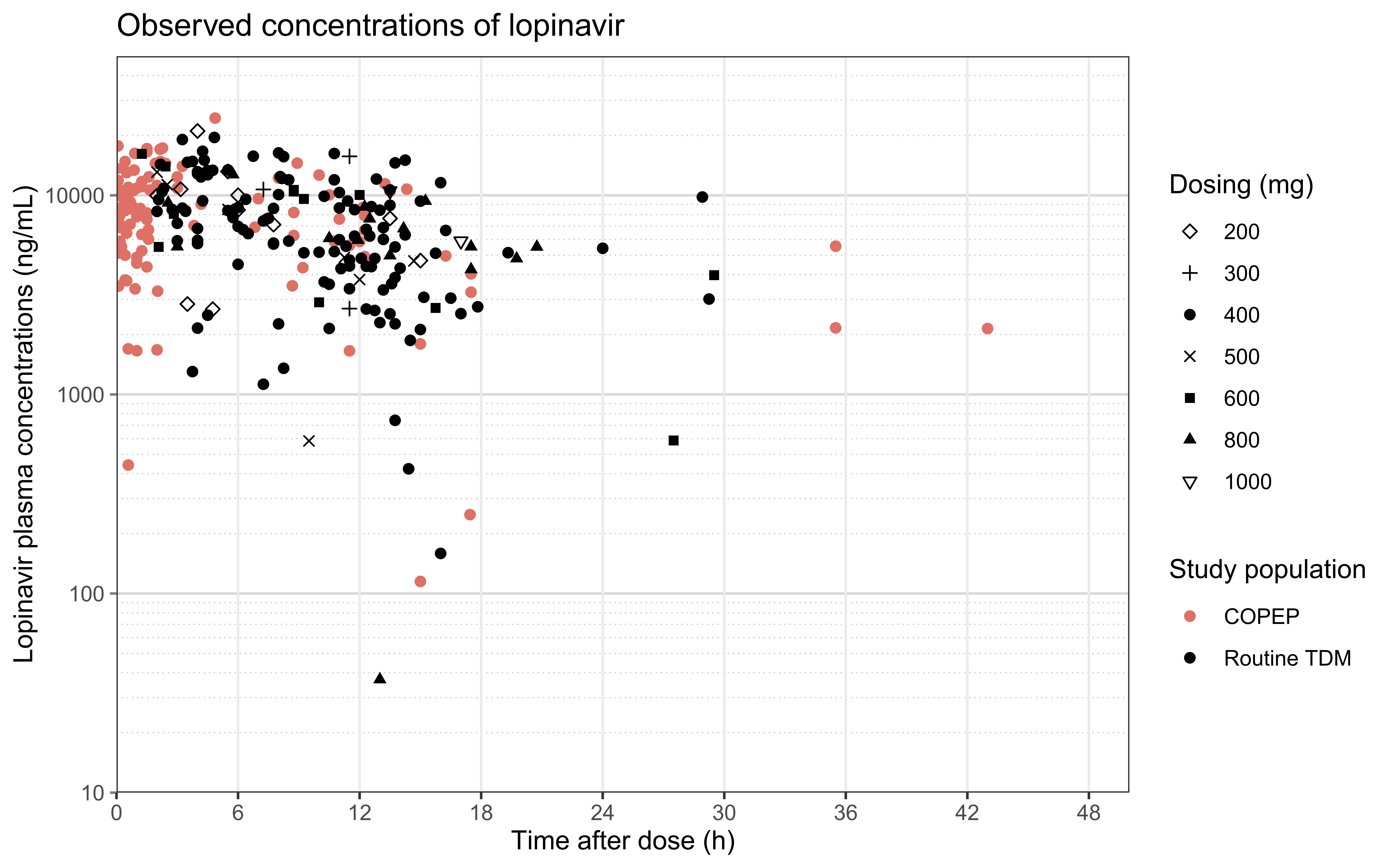


Figure S1: Observed plasma concentrations of LPV for the two study populations.


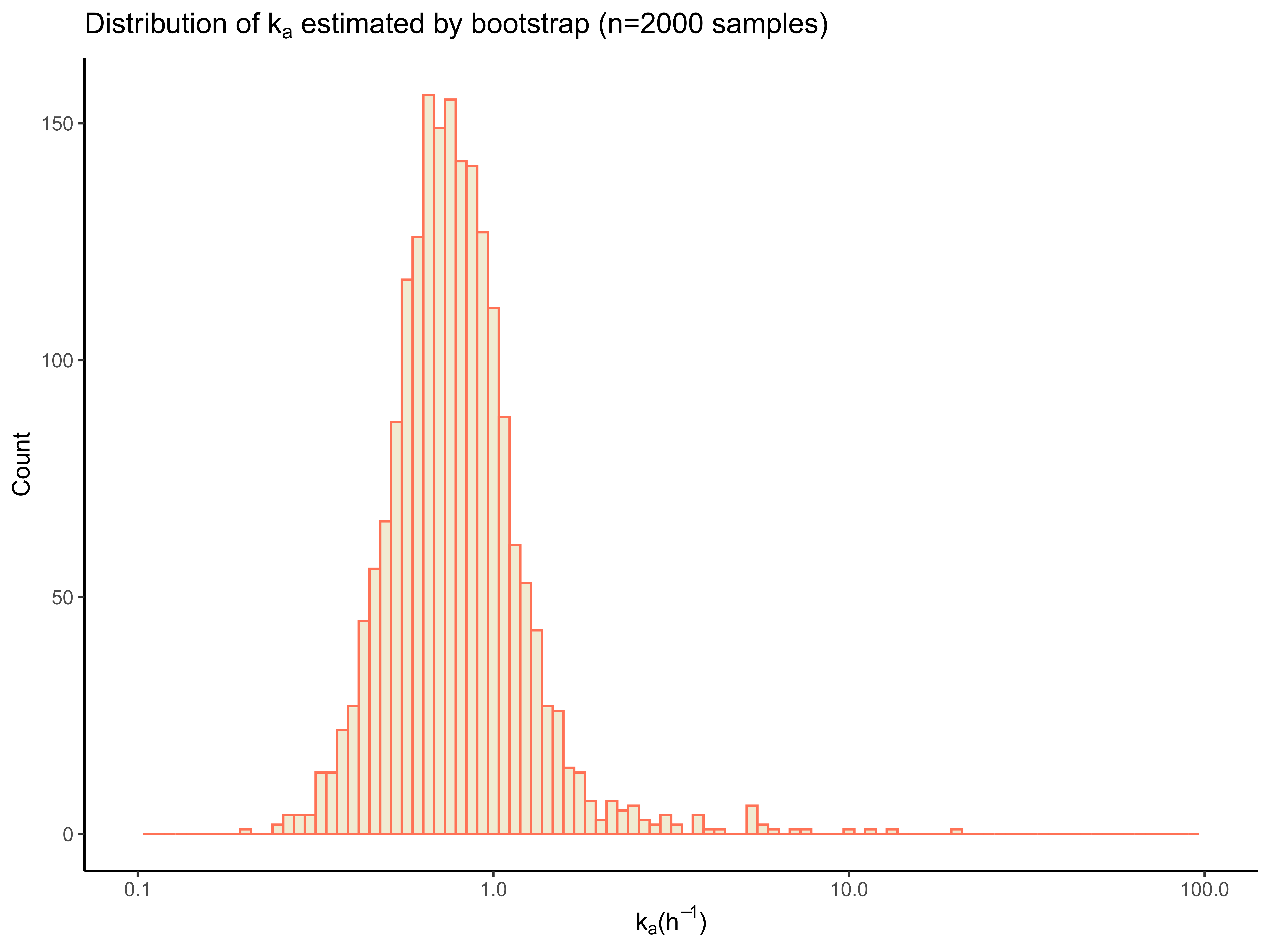


Figure S2: Distribution of k_a_ estimated by bootstrap.

Table S1: PK parameters of the published popPK models.

| **Models** | **Parameters (RSE, %)^a^** | | | | | | | | | **t_1/2_**  **(h)** |
| --- | --- | --- | --- | --- | --- | --- | --- | --- | --- | --- |
|  | **k_a_**  **(h^-1^)** | **IIV**  **(%)^b^** | **Lag time**  **(h)** | **V_LPV_**  **(L)** | **IIV**  **(%)^b^** | **CL_LPV_**  **(L/h)** | **IIV**  **(%)^b^** | **σ_prop_**  **(%)** | **σ_add_ (ng/mL)** |  |
| **In-house** | **0.743 (4)** | **-** | **-** | **78.9 (3)** | **-** | **4.05 (4)** | **30 (14)** | **32.8 (12)** | **1690 (1)** | **13.5** |
| Alvarez et al.^16^ | 0.572 | - | - | 94.8 | 80.1 | 4.88 | 288.1 | 18.6 | 71 | 13.5 |
| Niu et al.^17^ | 0.985 | - | 0.522 | 117 | - | 5.9 | 23.5 | 27.9 | - | 13.7 |
| Fuchs et al.^18^ | 0.42 | - | - | 62 | - | 4.4 | 31 | 35 | 1100 | 9.8 |
| Dickinson et al.^19^ | 0.26 | 29.2 | 1.7 | 14.9 | 28.2 | 4.1 | 23.1 | 42.9 | 2 | 2.5 |
| Lubomirov et al.^20^ | 0.3 | - | - | 70.8 | 134.2 | 5.72 | 36.1 | 26.3 | 2150 | 8.6 |
| Moltó et al.^21^ | 0.85 | - | - | 98.8 | 50 | 4.31 | 30 | - | 1900 | 15.9 |

k_a_: first-order absorption rate constant; V_LPV_: apparent volume of distribution of lopinavir; CL_LPV_: apparent clearance of lopinavir; σ_add_: additive residual error; σ_prop_: proportional residual error; t_1/2_: half-life.

^a^ Relative standard error (RSE) of the estimate defined as SE estimate/estimate, expressed as a percentage, with SE estimate retrieved directly from the NONMEM output file.

^b^ Inter-individual variability.
